# Increased stability of short femoral stem through customized distribution of coefficient of friction in porous coating

**DOI:** 10.1038/s41598-024-63077-w

**Published:** 2024-05-28

**Authors:** Konstantina Solou, Anna Vasiliki Solou, Irini Tatani, John Lakoumentas, Konstantinos Tserpes, Panagiotis Megas

**Affiliations:** 1 Department of Orthopaedic Surgery and Traumatology, School of Medicine, University of Patras, Patras, Greece; 2 Department of Mechanical Engineering & Aeronautics, University of Patras, Patras, Greece; 3 Department of Medical Physics, School of Medicine, University of Patras, Patras, Greece

**Keywords:** Short femoral stem, Porous coating, Optimized distribution, Stress shielding, Finite element analysis, Total hip arthroplasty, Biomedical materials, Implants

## Abstract

Stress shielding and aseptic loosening are complications of short stem total hip arthroplasty, which may lead to hardware failure. Stems with increased porosity toward the distal end were discovered to be effective in reducing stress shielding, however, there is a lack of research on optimized porous distribution in stem’s coating. This study aimed to optimize the distribution of the coefficient of friction of a metaphyseal femoral stem, aiming for reducing stress shielding in the proximal area. A finite element analysis model of an implanted, titanium alloy short-tapered wedge stem featuring a porous coating made of titanium was designed to simulate a static structural analysis of the femoral stem's behavior under axial loading in Analysis System Mechanical Software. For computational feasibility, 500 combinations of coefficients of friction were randomly sampled. Increased strains in proximal femur were found in 8.4% of the models, which had decreased coefficients of friction in middle medial areas of porous coating and increased in lateral proximal and lateral and medial distal areas. This study reported the importance of the interface between bone and middle medial and distal lateral areas of the porous coating in influencing the biomechanical behavior of the proximal femur, and potentially reducing stress shielding.

## Introduction

Total hip arthroplasty with short femoral stem is an effective treatment method for hip osteoarthritis, as they demonstrated better load in the proximal metaphyseal bone, improving proximal implant fixation and osseointegration^1–4^. Clinical studies using short femoral stems demonstrated promising short- and long-term results^1,5^. However, complications like hip pain, aseptic loosening, and stress shielding, leading to bone loss in the calcar and proximal lateral area of the femur were reported^6–12^. Although the primary stability of such stems is important until osseointegration, an average subsidence of the femoral stem ranged between 0.39–1.04 mm has been reported in short follow up^1,3,11^.

A meta-analysis prioritized short stems for middle-aged patients, and digital image correlation (DIC) and finite element analysis (FEA) suggested differences in load transfer and stress shielding effect^13^. Additionally, through DIC and FEA^8,14^, it was proposed that there is a difference in load transfer and stress shielding effect even between different short stems of the same category by Khanuja classification^3,15^. Different methods were proposed to address bone mass loss, cortical hypertrophy, and implant subsidence, limiting fracture and aseptic loosening risk either by modifying femoral stem geometry^16,17^ or material properties^18–22^.

Stems with different porosities were discovered to be effective in reducing stress shielding and delivering stable implant attachment through bone ingrowth^23,24^. Stems with increased graded porosity toward the distal end promoted osseointegration and reduced bone resorption^25,26^. Fully porous stems with axially graded stiffness were most advantageous for reducing stress shielding^27^. However, there is a lack of research on mechanical behavior of optimized porous distribution^23^.

The purpose of this study was to identify the characteristics of the porosity structure of a short metaphyseal femoral stem and to optimize the distribution of the coefficients of friction of the coating to design an optimized stem with a biomechanical behavior similar to physiological bone in the proximal area. The hypothesis was that the increase in porosity in the calcar area could decrease the stress shielding in the proximal femur.

## FEA model

In this study, 3D FEA models were created, consisting of a short-tapered wedge stem (TRI-LOCK Bone Preservation Stem, DePuy Orthopaedics Inc. Warsaw, IN, USA) made of titanium alloy (Ti6Al4V) with a highly porous coating (“GRIPTION®”) on the proximal 50% portion, implanted in a femur model using CAD software. Its porous coating features an average pore size of 300 μm, which lies within the optimal range for tissue growth into the structure and enables vascularization; an average volume porosity 63% and coefficient of friction (CF) equal to 1.2.

**Figure 1:**
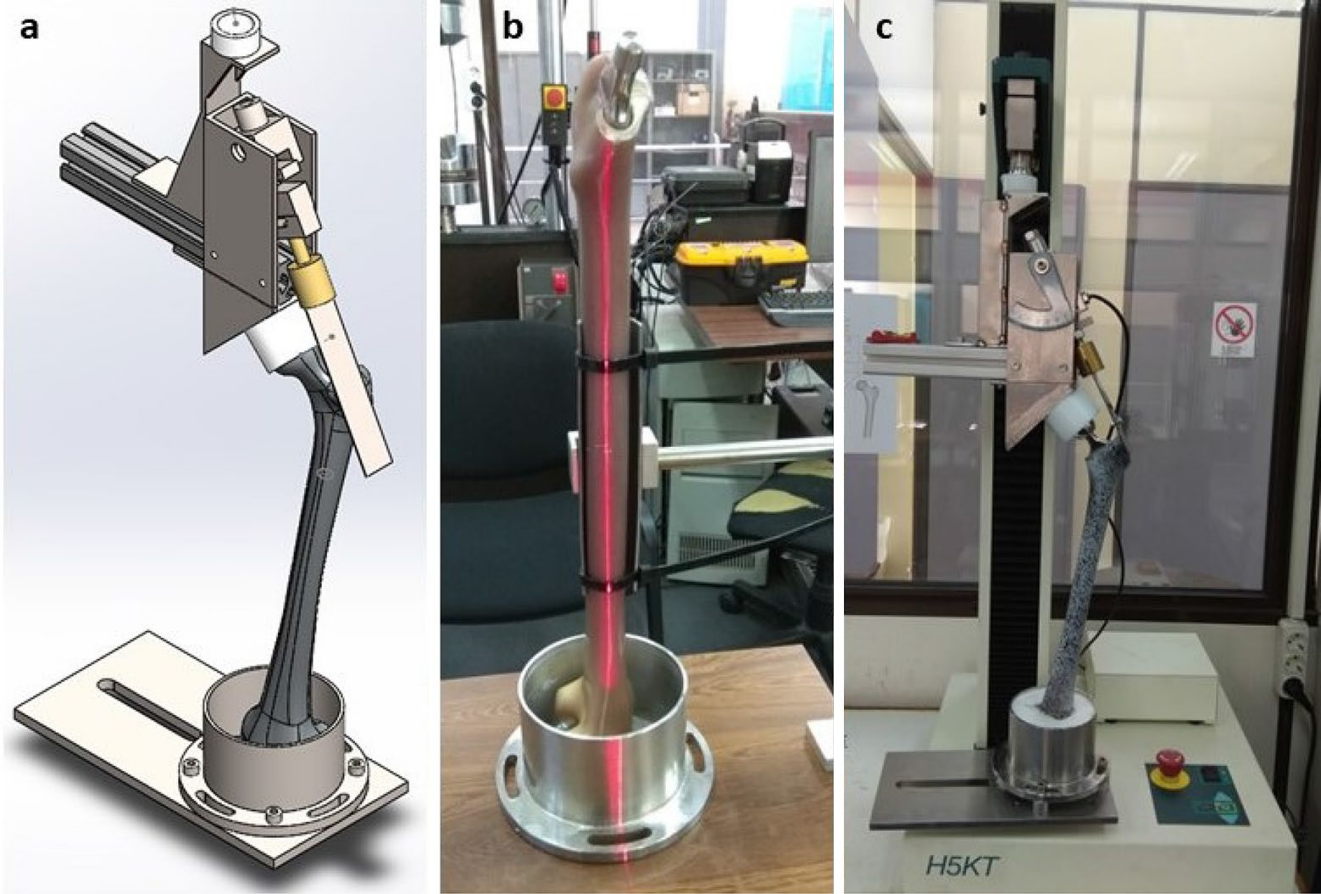
(**a**) Layout of the axial loading experiment of an implanted prothesis in femur (previously published^41^); (**b**) Establishment of femoral axis before stabilizing the femur with epoxy distally for the experimental biomechanical study; (**c**) Loading system configuration (direction of hip and abductors’ forces).

Our study utilized cortical and cancellous bone material with Young's modulus of elasticity E = 16.7GPa and E = 155 MPa, based on previous data and sawbones’s manufacturers' suggestions^28–32^. This study was based on previous published experimental results from an axial load test and validated the FEA model using cortical surface-strain distribution in intact and implanted femurs^14,17^ (Fig. 1). Strains were examined both macroscopically and microscopically, and statistical comparisons were conducted among the experimental and FEA models. Comparison through Mann–Whitney/Wilcoxon Rank-Sum demonstrated no statistical differences in the distributions of strains in lateral (p = 0.443) and in medial (p = 0.160) cortex between experimental and FEA model, hence the FEA model results coincided to the DIC ones (Supplementary material 1). Verification of consistency and accuracy was assessed by running each simulation twice.

**Figure 2:**
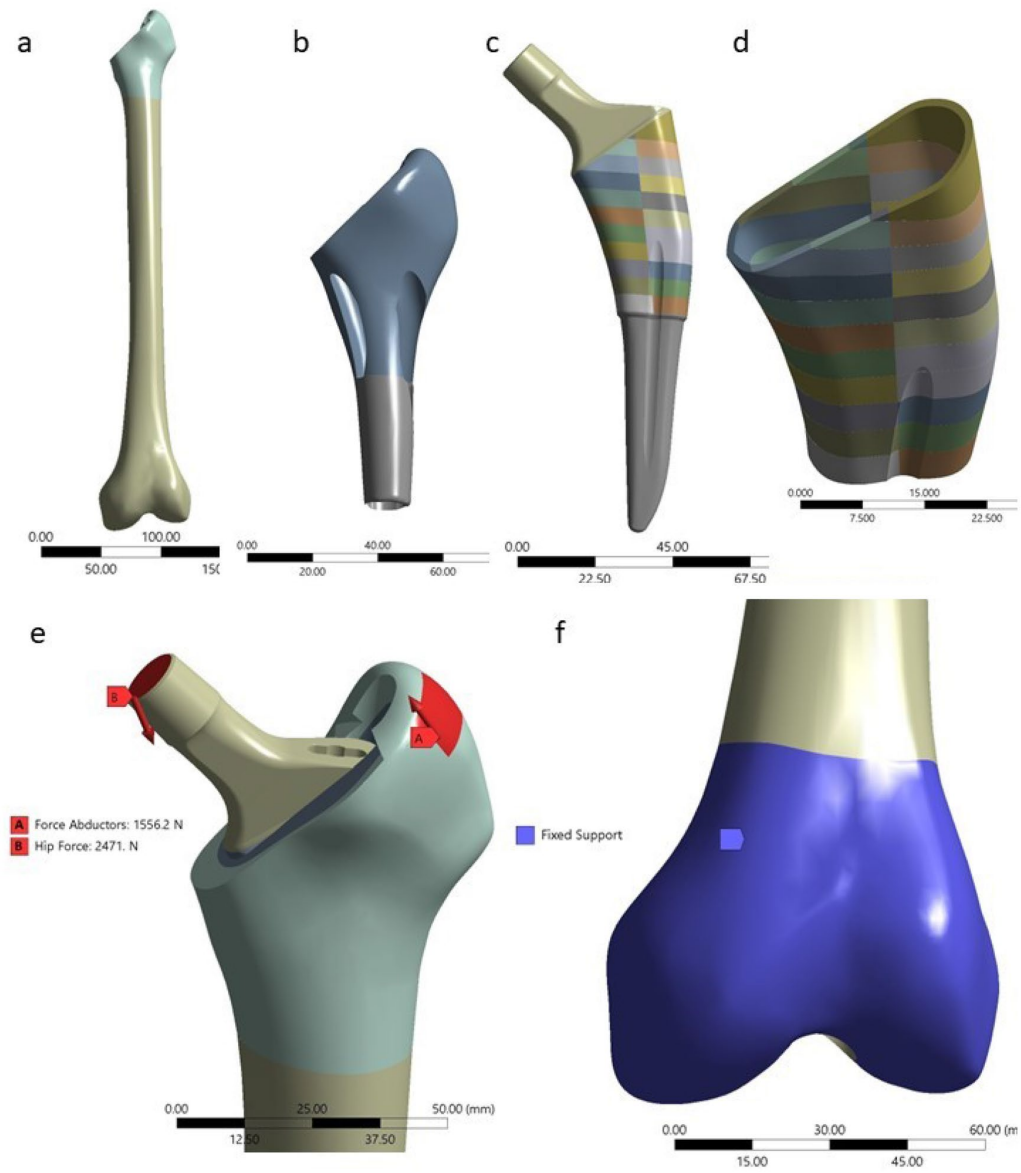
Geometry of the computational model. (**a**) femoral part consisting of cortical bone; (**b**) proximal femoral area consisting of cancellous bone; (**c**) femoral prothesis; (**d**) porous coating part of the stem; (**e**) loading system configuration (force applied by abductors and hip force); (**f)** system’s boundaries (fixed support of the distal femoral part).

The Analysis System (ANSYS) Mechanical Software was used for the simulation. The implant’s geometry, including the porous coating part was acquired from computed tomography imaging (DICOM files), and converted into .STEP files, with minor modifications in its geometry for adjusting the porous coating for FEA analysis (Fig. 2). The coefficient of friction, influenced by surface roughness, lubrication conditions, temperature, contact pressure; indirectly influences the porosity of Ti6AI4V^33–35^. We used as constants the geometry design of Tri-Lock BPS short stem and the material Ti6Al4V alloy and we examined as variable the distribution of the coefficient of friction of the porous coating under FEA analysis of axial load.

In ANSYS Workbench 2020 (R2), bone and stem geometries were set in space planes, and material properties were assigned. Contact interfaces between cortical and cancellous bone parts, and between solid stem parts and porous coat were set as bonded; loads were transferred between bonded components through their common interface, and components would never be separated from each other. The interfaces between bone parts and solid stem parts were set as frictionless due to the smooth area of the solid part; however, the contact interfaces between bone parts and porous coat parts; were assigned as frictional based on Coulomb’s Law of Friction.

$${F}_{f}riction \le mu* {F}_{n}ormal$$

where, ${F}_{f}riction$ is the frictional force; mu (μ) is the coefficient of friction; ${F}_{n}ormal$ is the normal force.

The contact parameters were set as the surfaces between the contact body and the target body, the Augmented Lagrange formulation, the penetration tolerance of 10% of underlying elements depth, elastic slip tolerance of 1% of average contact length in pair and normal stiffness equal to 10. The analysis solver was set to detect any deflection and the full Newton–Raphson method was used to update solution until convergence.

During ipsilateral single-limb stance it has been found that the joint-contact force was 2.1 times body weight, and during the stance phase of gait the peak force typically was 2.6 to 2.8 times body weight, allowing the assessment of the structural integrity and loading patterns of the femur under weight-bearing conditions^36^. During the single leg stance, adductor muscles resist the torques produced by the body weight in order to maintain stability^37^. Cristofilini et. al.^38^ presented that the applied hip force should be around 29° to the femoral diaphysis and the abducting force around 40° to the femoral diaphysis. The angle between the joint reaction force combined with the abductor force at the center of the femoral head and the human body’s partial gravity and the vertical line on the ground is 9–12 degrees^10^. Hence, the study replicated the natural inclination of single-leg stance by fixing the distal femoral end neutral on the sagittal plane and at 11 degrees of adduction in the frontal plane. We applied a hip force of 2471N at 29° to the femoral diaphysis and an abducting force of 1556 N at 40° to the femoral diaphysis based on previous published experiment^14^ (Fig. 2e,f).

**Figure 3:**
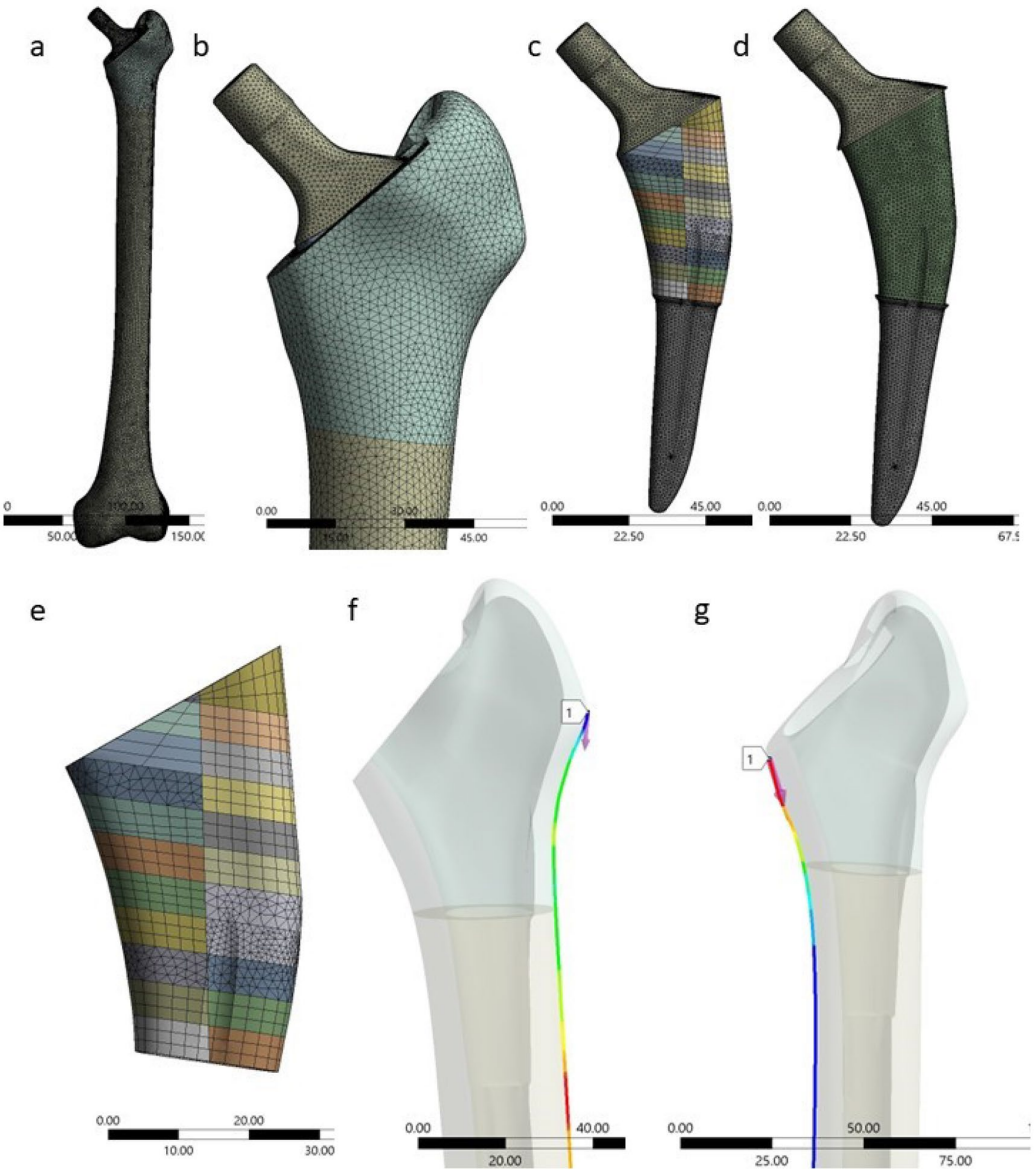
Mesh construct (**a**) of the implanted stem in femur; (**b**) of the proximal part of the femur; (**c**) of the entire prothesis; (**d**) of the solid part of femoral stem; (**e**) of the porous coating part. Paths designed in lateral (**f**) and medial (**g**) cortex to measure strain distribution.

FEA discretization was performed using meshes of over 2,196,924 nodes of tetrahedral or hexahedral elements per model. Body sizing was used to refine the mesh in certain regions and to apply grading for smoother transitions. The edge length was 2 mm for cancellous and cortical bone interfaces and 0.5 mm for porous coat and bone interfaces, which is known from previous studies to be sufficient for mesh convergence and fine material distribution that can differentiate cortical and cancellous bone areas^39,40^. Face sizing was used to manage mesh density, element size on surfaces and refine mesh in areas with intricate geometry and bone components, resulting in 1,526,318 elements, aspect ratio 1.8506 ± 1.3451 and element quality 0.84239 ± 0.10224 (Fig. 3).

**Figure 4:**
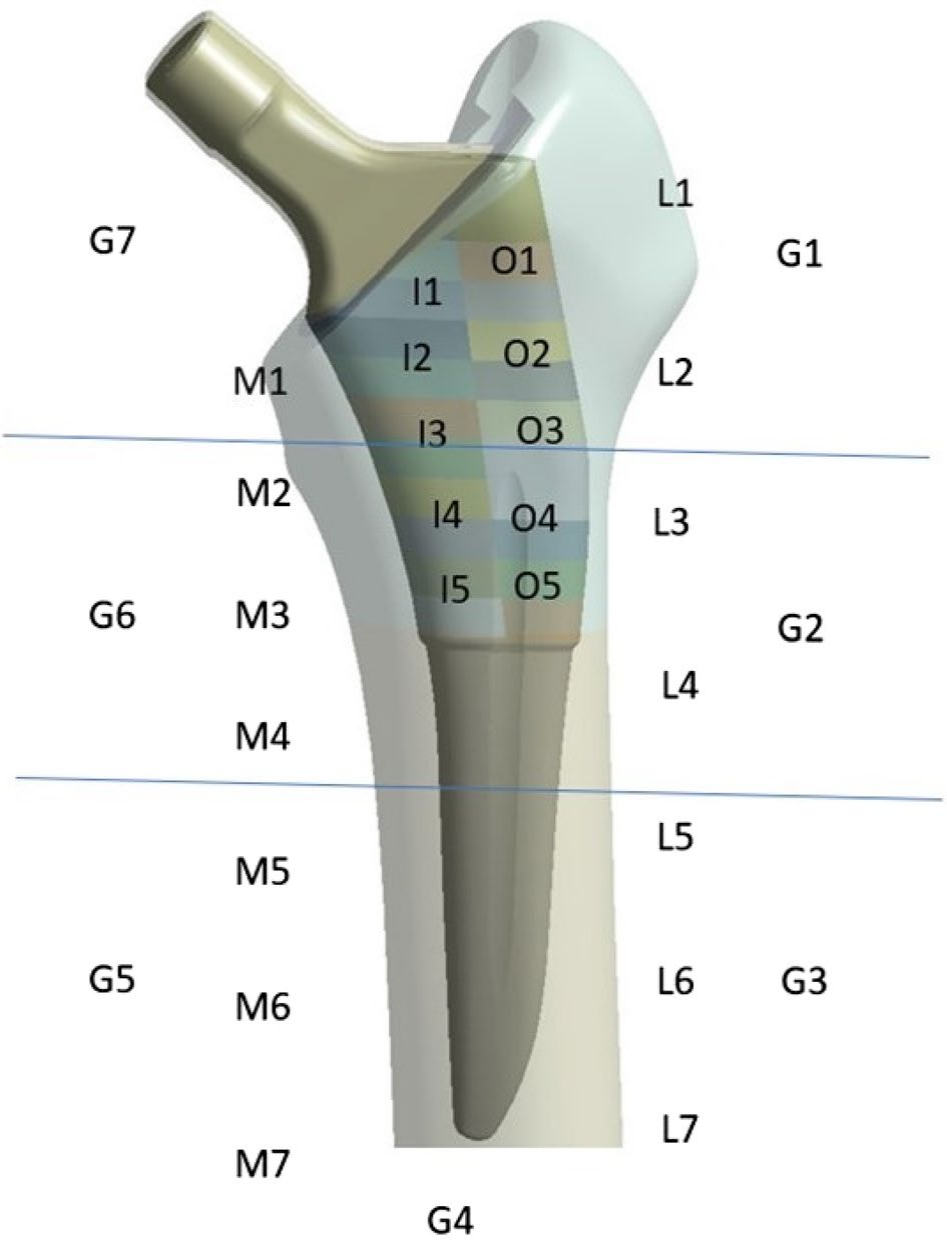
Porous coating medial (I1, I2, I3, I4, I5) and lateral areas (O1, O2, O3, O4, O5). Medial (M1, M2, M3, M4, M5, M6, M7) and lateral (L1, L2, L3, L4, L5, L6, L7) path measurements. Gruen zones (G1, G2, G3, G4, G5, G6, G7).

A static structural nonlinear analysis was used to simulate the femoral stem's behavior under axial loading, providing information on strain distribution, displacement and deformation. Biomechanical studies of proximal femur proposed that the difference of the surface strain is an appropriate proxy for stress shielding^20^. Thus, two paths were designed for measurements, with principal maximal and minimal strains measured in the medial and lateral cortex, respectively (142 points each) (Fig. 3f,g). The measurements in the two paths were separated every 20 mm until a maximum of 40 mm in the proximal medial and lateral areas {M1, M2, L1, L2}, respectively, which corresponded to Gruen zones 1 and 7 (Fig. 4)^42^.

Ti6AI4V has a moderate friction coefficient in dry conditions, falling between 0.4 and 0.6. The current market model, “model zero”, has a friction coefficient of 1.2 and a relative density of 63% of the porous coat. So, friction coefficients between 0.5 and 1.5 were used in research models. The porous coat was separated into 10 areas of height 2 mm in the medial (I1, I2, I3, I4, I5) and lateral (O1, O2, O3, O4, O5) (Fig. 4). Each of them could take seven values in the domain {0.5, 0.7, 0.9, 1.1, 1.2, 1.3, 1.5}, but the O1 was decided to remain stable (CF = 1.2) due to the complex geometry and meshing of that area of the porous coat and due to the fact that in the experiment a metallic rod was attached to the lateral aspect of the greater trochanter, using epoxy glue, to simulate the hip abductor muscles, so measurements could not be taken into account. All models were compared with “model zero”, which was defined as the model with CF = 1.2 in all areas of the porous coat.

### Statistical analysis

The discrete set contained 7 values {0.5, 0.7, 0.9, 1.1, 1.2, 1.3, 1.5} assigned to 9 variables {O2, O3, O4, O5, I1, I2, I3, I4, I5}, with O1 remaining stable, creating a grid of 7^9^ nodes (more than 40 million). The FEA model's complexity made it impossible to run all combinations. To ensure computational feasibility, 500 combinations were randomly sampled without replacement, and conditions were met for the experiment to be accurate due to common summary statistics (means, medians, quartiles) and no strong correlations between the nine variables, mutually pairwise. Data analysis was performed in R και RStudio.

Each experiment resulted in a set of values that defined a non-parametric distribution (according to the Shapiro–Wilk test) requiring non-parametric descriptive and inferential statistics. Wilcoxon's signed-rank test was used to compare the paired values of each of the areas M1, M2, L1 and L2, while Spearman's correlation test assessed monotonic relationships between femur areas and friction coefficients. Differences (paired) were expressed as the median of subtracting the “model zero” values minus the examined model values.

The absolute correlation ranged from very weak (0–19%) to moderate (40–59%) to strong (60–79%), with Spearman's correlation being most significant at 80–100%. In our 500 samples, an absolute correlation of more than 8.5% was defined as statistically significant. Regression analysis examined the relationship between the independent variables of the coefficient of friction and the dependent variable of each femoral area and defined the way in which combined changes influenced each outcome. Lastly, Poisson count regression was utilized to predict the increased strains in areas M1, M2, L1, L2 simultaneously (number of satisfied conditions could be in {0, 1, 2, 3, 4}), within the combinations of friction coefficients.

## Results

We analyzed 500 different combinations of coefficients of friction around the porous coating area. The FEA demonstrated that 42 models (8.4%) showed increased strains in the proximal areas M1, M2, L1 and L2 compared to model zero. Twelve out of them (28.5%) had a statistical increase compared to the model zero of the order of 10 to the power of minus seven or eight (mm) (p < 0.05).

**Figure 5:**
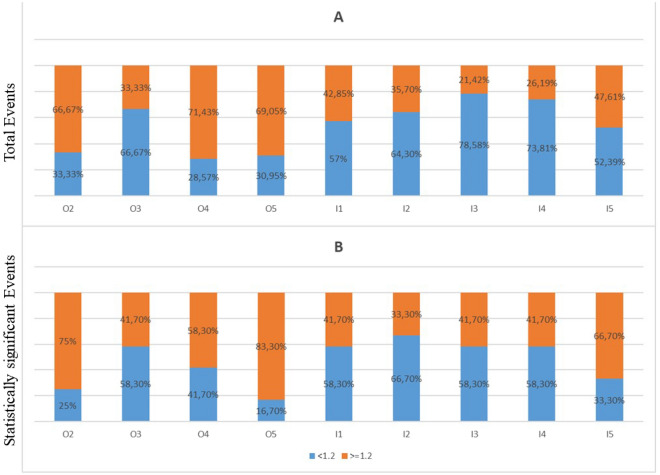
(**a**) Chart of increase or decrease in each porous coating area (O2, O3, O4, O5, I1, I2, I3, I4, I5) for the 42 models, that achieved increased strains in proximal areas; (**b**) Chart of increase or decrease in each porous coating area (O2, O3, O4, O5, I1, I2, I3, I4, I5) for the 12 models, that achieved statistical increase in strains in proximal areas (p < 0.05).

In the majority, there was a decrease in the coefficient of friction in the medial middle distal areas of the porous coating (I2, I3, I4) and in O3, which resulted in an increase in proximal femoral strains. Moreover, it was observed that the strains in proximal areas increased, when the coefficient of friction in the areas O2, O4, O5 of the porous coating was greater than 1.2 (Fig. 5).

**Figure 6:**
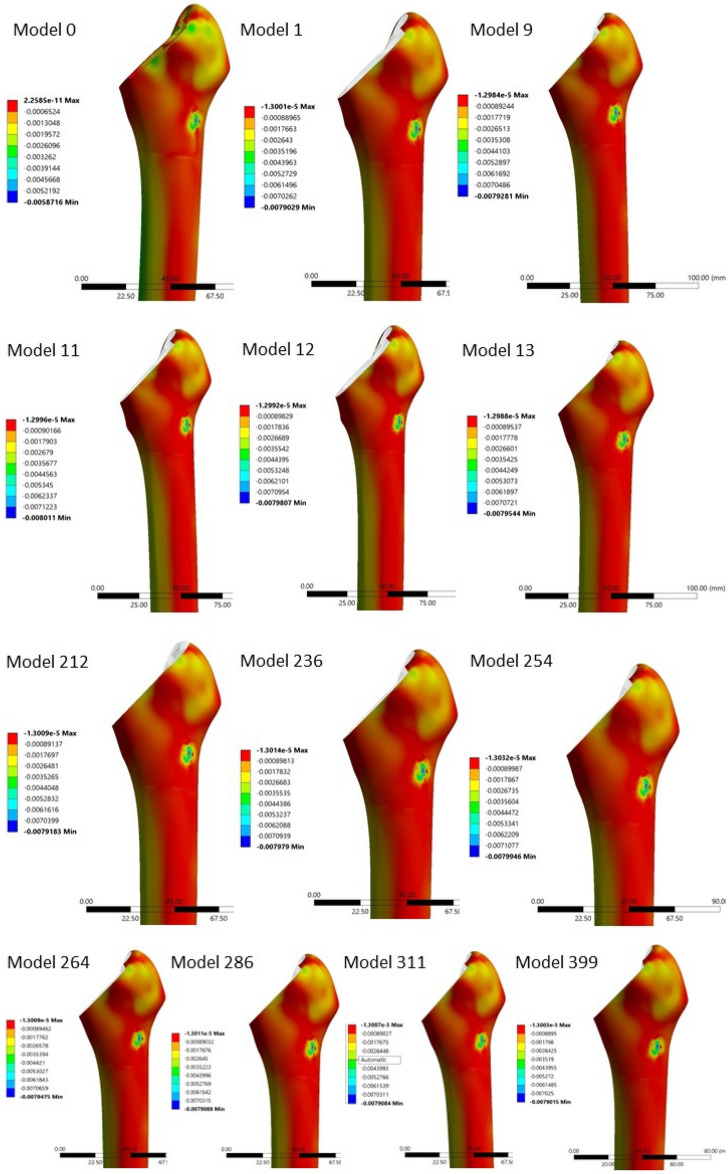
The minimal principal strains in “model 0” and the 12 models with statistical increase in strains in all four proximal areas (M1, M2, L1, L2).

**Figure 7:**
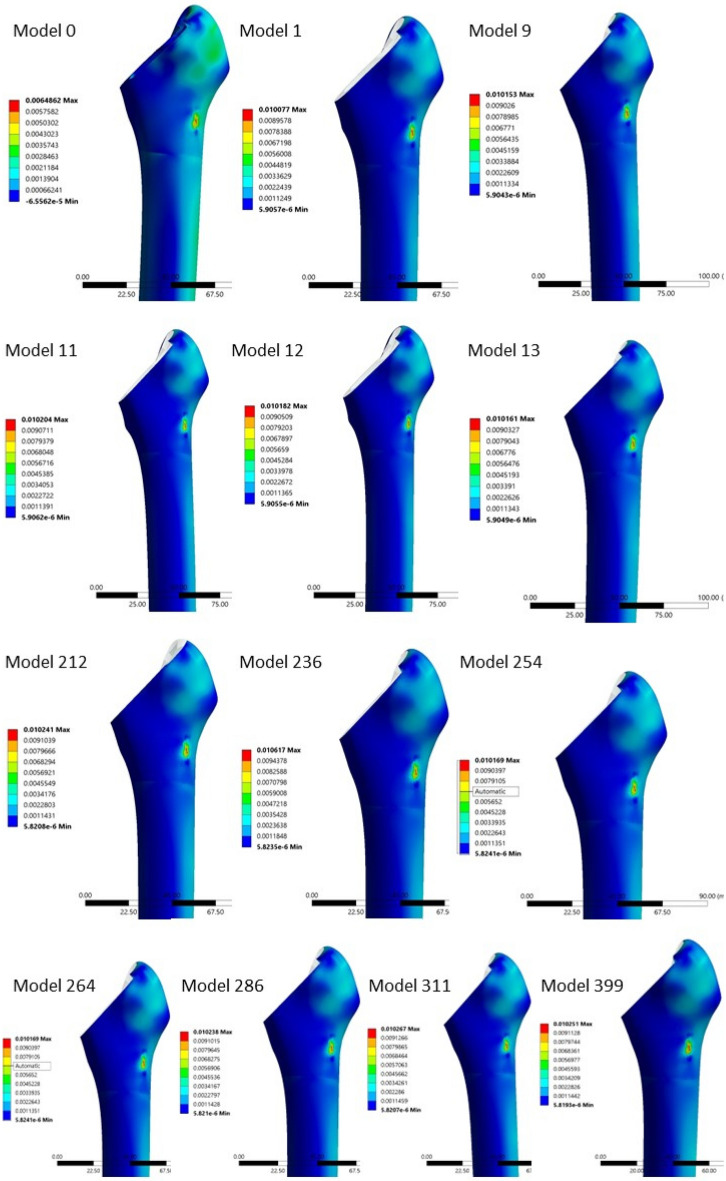
The maximal principal strains in “model 0” and the 12 models with statistical increase in strains in all four proximal areas (M1, M2, L1, L2).

Between the twelve models with statistical increases (p < 0.05) in the proximal areas’ strains, two groups were identified (Figs. 6 and 7). The first group had a coefficient of friction of more than 1.2 in the area O2 and in the areas I1, I2—except the model N1, which had 0.9. The rest of the areas had a friction coefficient of 1.2, while the O3 was varying. The second group had a coefficient of friction less than 1.2 in all medial areas (I1, I2, I3, I4, I5) and in O4 and an increased coefficient of friction in O5 (Table 1). Both groups result in increased strains in proximal medial and lateral areas M1, M2, L1 and L2. Increasing the coefficient of friction in proximal areas (O2, I1 and I2) resulted in increased contact forces between porous coating and surrounding bone, which led to higher strains. Decreasing the coefficient of friction in mid and distal porous coat, resulted in decreased forces between porous coat and surrounding bone in these areas, which led to lower strains, thus load was transferred to the more proximal areas, where the strains were increased.

**Table 1:**
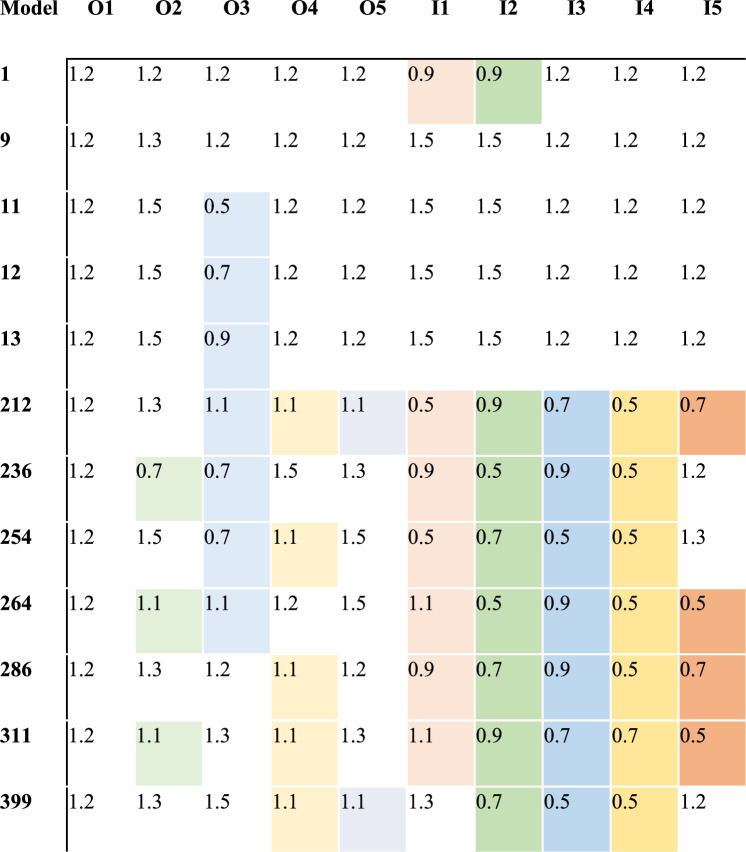
Models (rows) with their combinations of coefficients of friction O1, O2, O3, O4, O5, I1, I2, I3, I4, I5 (columns) had statistical differences (*P* < 0.05) in all proximal areas (M1, M2, L1, L2).

*Color means that the value is less than 1.2.

Spearman's correlation analysis had unveiled relationships within the proximal femur's medial region. Notably, M1 exhibited a moderately positive correlation with the coefficients of friction in I3 (57.54%) and I4 (52.52%). Furthermore, in the proximal femur's M2 area, there was a strong positive correlation with I3 (77.31%) and a moderate negative correlation with I5 (-43.57%). On the lateral proximal area, L1 exhibited a strong negative correlation with the coefficient of friction in O4 (-85.24%) and O5 (-60.95%). L2 showed a strong positive correlation with O4 (71.89%) and a moderate correlation with O2 (47.61%). Notably, all the other areas of coefficients of friction showed weak correlations with each proximal femoral area (Table 2). The graphs of Spearman's correlation are shown in Supplementary Material 2–5.

**Table 2:** Spearman’s correlation analysis for coefficients of friction and differences in strains in proximal areas.

	M1 (%)	M2 (%)	L1 (%)	L2 (%)
O2	− 39.51%	− 12.95	− 15.34	**47.61**
O3	− 25.77%	− 3.57	11.99	39.43
O4	− 26.79%	4.69	**− 85.24**	**71.89**
O5	− 18.24%	6.71	**− 60.95**	7.12
I1	− 33.81%	− 6.31	− 0.07	26.29
I2	− 13.15%	9.99	6.97	29.95
I3	**57.54**	**77.31**	9.24	4.34
I4	**52.62**	34.71	− 1.13	22.41
I5	− 18.35	**− 43.57**	1.16	23.48

Strong or moderate correlations are in bold. Notice that an absolute correlation of more than 8.5% is statistically significant in the given sample.

The regression analysis results are presented in Table 3. Homogeneous results were demonstrated for increases in O5 and decreases in I2, I3 and I4 in order to increase strains in proximal areas. While in O2, O3, O4, I1 and I5, there was no consensus between femoral areas. Poisson count regression analysis that targeted the number of successful (increased strains in M1, M2, L1 and L2) demonstrated a statistical decrease in the coefficients of friction in areas I3 and I4 and a statistical increase in O2, O5 and I5.

**Table 3:** Regression analysis of independent variables O2–O5 and I1–I5, and dependent variables M1, M2, L1, L2, expressed by difference between ${M1}_{model0}-{M1}_{modelX}$.

Areas of porous coating	Difference M1		Difference M2		Difference L1		Difference L2		Increased strains in all proximal areas	
	Estimate (*10^−5^)	*P*-value*	Estimate (*10^−5^)	*P*-value*	Estimate (*10^−5^)	*P*-value*	Estimate (*10^−5^)	*P*-value*	Poisson Estimate	*P*-value*
O2	− 0.47	** < 0.001**	− 0.02	0.439	− 0.47	** < 0.001**	2.49	** < 0.001**	0.24	**0.025**
O3	− 0.39	** < 0.001**	0.06	0.058	0.15	** < 0.001**	1.94	** < 0.001**	− 0.11	0.366
O4	− 0.48	** < 0.001**	0.21	** < 0.001**	− 1.43	** < 0.001**	4.76	** < 0.001**	− 0.06	0.597
O5	− 0.27	** < 0.001**	0.05	0.074	− 0.69	** < 0.001**	− 0.75	** < 0.001**	0.43	** < 0.001**
I1	− 0.34	** < 0.001**	− 0.04	0.118	0.17	** < 0.001**	− 0.11	**0.04**	0.11	0.294
I2	0.06	0.138	0.38	** < 0.001**	0.18	** < 0.001**	0.20	** < 0.001**	− 0.03	0.775
I3	0.96	** < 0.001**	1.42	** < 0.001**	0.05	**0.010**	1.12	** < 0.001**	− 0.96	** < 0.001**
I4	1.22	** < 0.001**	0.59	** < 0.001**	− 0.02	0.372	0.96	** < 0.001**	− 0.41	** < 0.001**
I5	− 0.22	** < 0.001**	− 1.00	** < 0.001**	0.09	** < 0.001**	0.51	** < 0.001**	0.32	**0.018**

Poisson’s count regression analysis of successful conditions (increased strains in M1, M2, L1, and L2) and coefficients of frictions.

*P-value is significant when it is ≤ 0.05 (bold).

## Discussion

This study found that 8.4% of FEA models showed increased strains in proximal areas compared to a short tapered-wedge stem with a porous coat with a coefficient of friction of 1.2. The decrease in friction coefficients in I3 and I4 and the increase in O2, O5 and I5 resulted in reduced stress shielding proximally. The hypothesis that an increase in friction in the proximal medial area of the calcar could reduce stress shielding was partially verified by the first group of five models. However, the statistical analysis did not confirm the hypothesis about the porosity of the calcar area, as reduced friction in middle medial and distal porous coat areas was correlated with reduced stress shielding.

This analysis had limitations due to differences between simulation and clinical environments. Models were run twice for verification and interobserver consistency was 100%. The study did not investigate the dynamic loading stimulus or clinical response of a 3D printed porous stem implanted into bone, which is part of a future project. The discrete and optimized porosity distribution on femoral stems discussed in this research may differ from graded porous stems with continuous distribution presented in the literature^23^, but it appeared to perform better biomechanically. Another limitation was that we performed a simulation by simplifying the model for computational efficiency, however further research should be conducted with multi-scale models to investigate the load transfer patterns and bone growth. In this study, we investigated the distribution of coefficient of friction in the coating of a short tapered femoral stem made by Ti6Al4V, however future research should focus on establishing a relationship between porosity and coefficient of friction for Ti6Al4V through experimentation or simulation on microscale.

Another limitation of this study was the use of sawbones’s cortical and homogenous cancellous properties instead of the heterogenous properties of real femur, in order to simplify the FEA simulation. Printing the optimized models and testing them in real femurs is part of future work, aiming to optimize the porous coating properties based on individual’s bone properties. The model design had limitations due to mesh density heterogeneity and the use of tetrahedral and hexahedral elements due to complex interfaces in porous coat proximal areas. The way that the contact parameters influenced the results, required further thorough parametric studies. As the FEA models had to be validated by the experimental technique, only surface strains were recorded. The study of the very-proximal lateral area L1 and the coefficient of friction in O1 was limited as the area of the great trochanter had contact inconsistence between elements due to a specific lamina in the experiment. Further research on distal Gruen zones and the entire femur is needed to draw safe conclusions.

To the best of our knowledge, no other study in existing literature researched the design of optimized non-graded porous coatings in short tapered-wedge stems^23,25,27,43^. Optimized porous coatings of Ti6Al4V with different coefficients of friction in local areas are possible to be produced by electron beam melting or by controlling the amount of powder being deposited or adjusting the energy input during the additive manufacturing process^44,45^. Increased strains in the proximal areas were achieved by 28.5% of the models, indicating the need for optimization in femoral prothesis for better mechanical performance^23^. However, group one showed that increasing the coefficient of friction proximally in the existing in market porous coat could reduce the stress shielding proximally, which was noted by clinical and biomechanical studies in short tapered-wedge stems^1,5,12^. On the other hand, the second group of seven stems showed increased strains in the proximal femur with decreased porosity in all medial areas. Similarly, graded stems with increased porosity inwardly showed the lowest bone density loss and lower peak micromotion, inducing osseointegration^25^. Moreover, the friction in the I5 and O5 distal areas was increased, performing biomechanically better proximally. Limmahakhun et. al. found that axially graded cellular structures reduced peri-implant stress shielding, potentially reducing revision surgeries^26^.

This study presented the importance of the interface between bone and the middle and distal areas of the porous coat in influencing the biomechanical behavior of the proximal area, which is in line with the mode of fixation of meta- diaphyseal stems with porous coating, where biologic fixation is therefore required in both the metaphysis and the diaphysis to achieve the proper amount of fixation at each level in order to allow transitional stress transfer^46^. Increased porosity distally was found to reduce stress shielding^27^, while our study confirmed that lower friction medially middle distal and higher friction lateral distally led to better biomechanical behavior proximally. However, this research was limited to proximal Gruen zones.

## Conclusions

The analysis revealed two types of coefficients of friction combinations that could prevent stress shielding proximally; either by reducing friction in medial areas and raising it lateral distally, or by increasing friction proximally and keeping it at 1.2 in middle distal porous coat areas. This partially validated the hypothesis that the calcar area's coefficient of friction influences strain distribution. It was demonstrated that the biomechanical behavior in the proximal femoral area was influenced by friction in the middle medial areas (I3, I4) and increased friction in the distal porous coating areas (I5, O5). Future research should focus on personalizing stems, considering the individual bone variations, in order to create an interface between bone and stem, which could create a better biomechanical environment.

### Supplementary Information

Supplementary Information 1.

Supplementary Information 2.

Supplementary Information 3.

Supplementary Information 4.

Supplementary Information 5.

Supplementary Information 6.

## Data Availability

The manuscript does not have associated data in a data repository due to the large volume of file storage. Available upon reasonable request. For further information, please contact the corresponding author at the following email address: k.solou@gmail.com.
