# Supplementary figures and images for: Increased stability of short femoral stem through customized distribution of coefficient of friction in porous coating
Source: Sci Rep. 2024 May 28;14:12243. doi: 10.1038/s41598-024-63077-w (PMC11133419; doi:10.1038/s41598-024-63077-w)

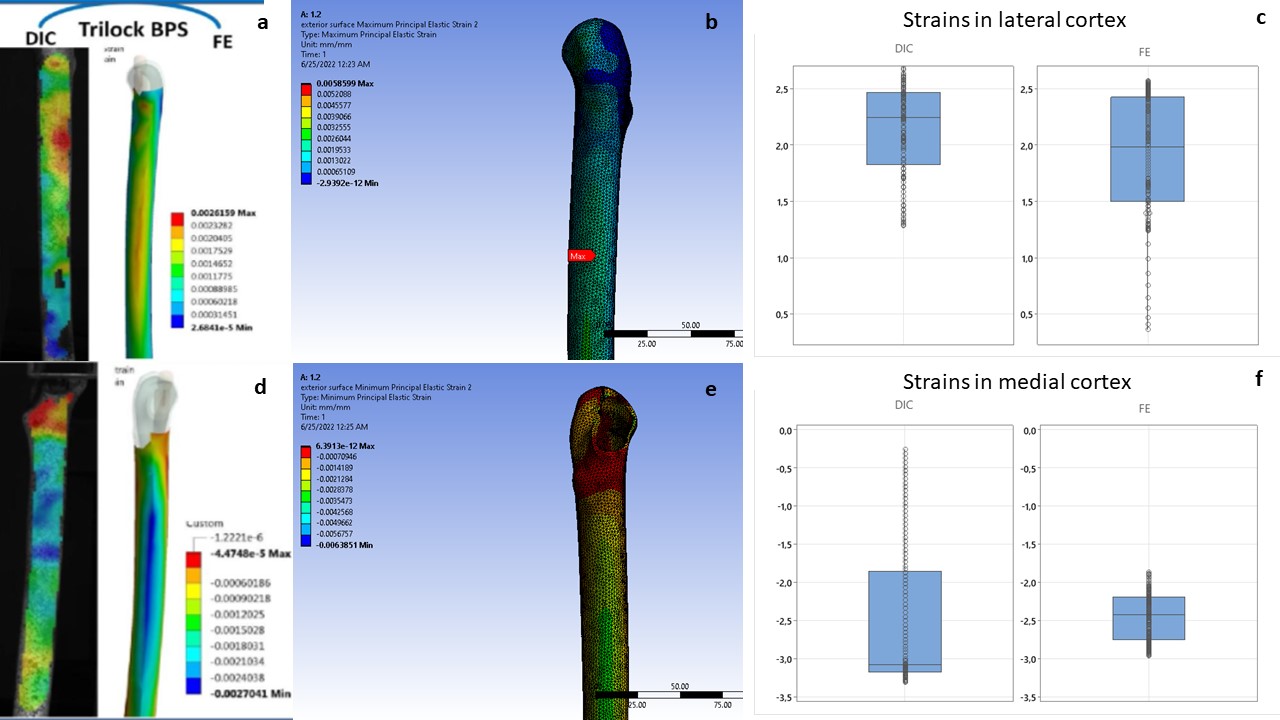

Supplement: Supplementary file 1 — Supplementary Information 1. [file 41598_2024_63077_MOESM1_ESM.jpg]

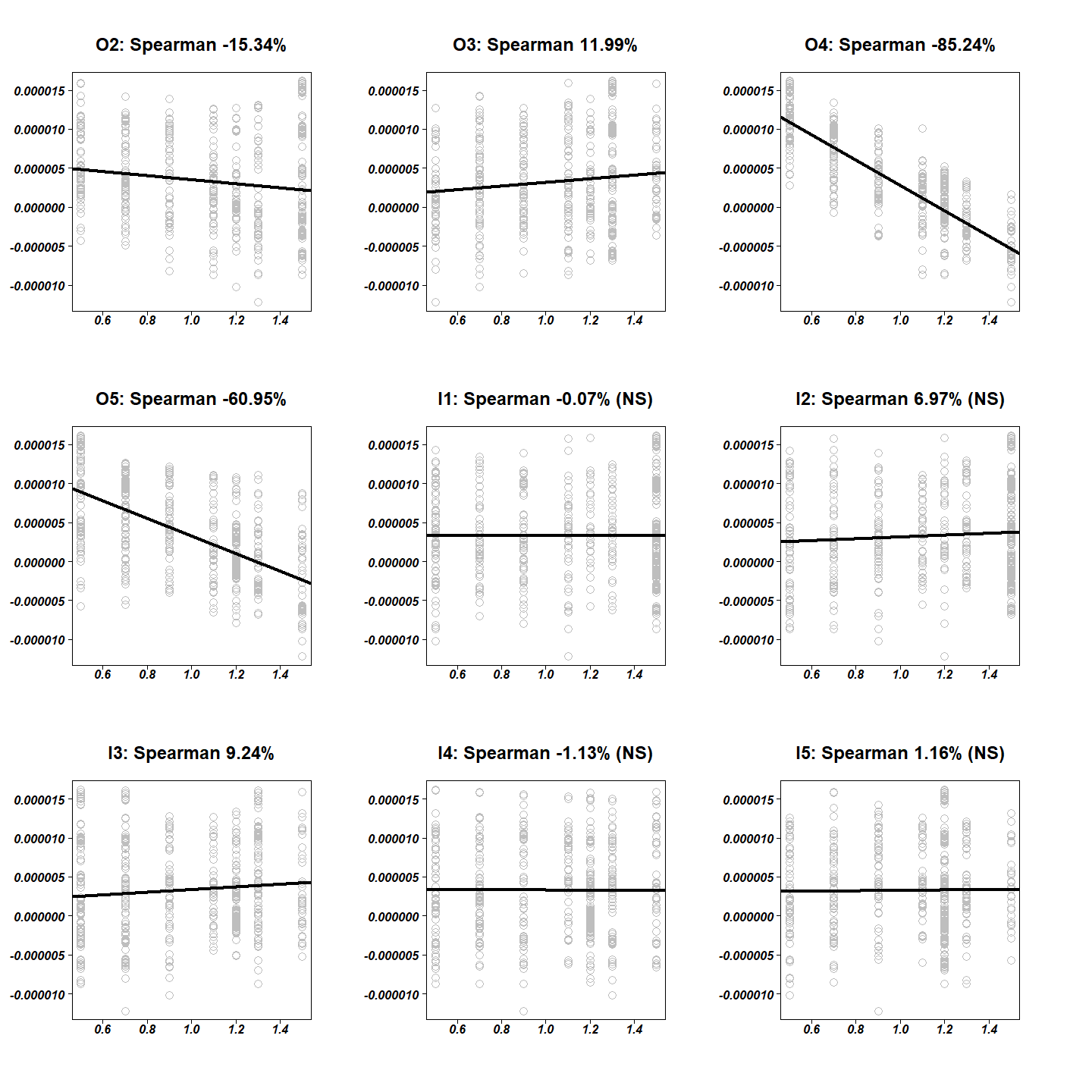

Supplement: Supplementary file 2 — Supplementary Information 2. [file 41598_2024_63077_MOESM2_ESM.tiff]

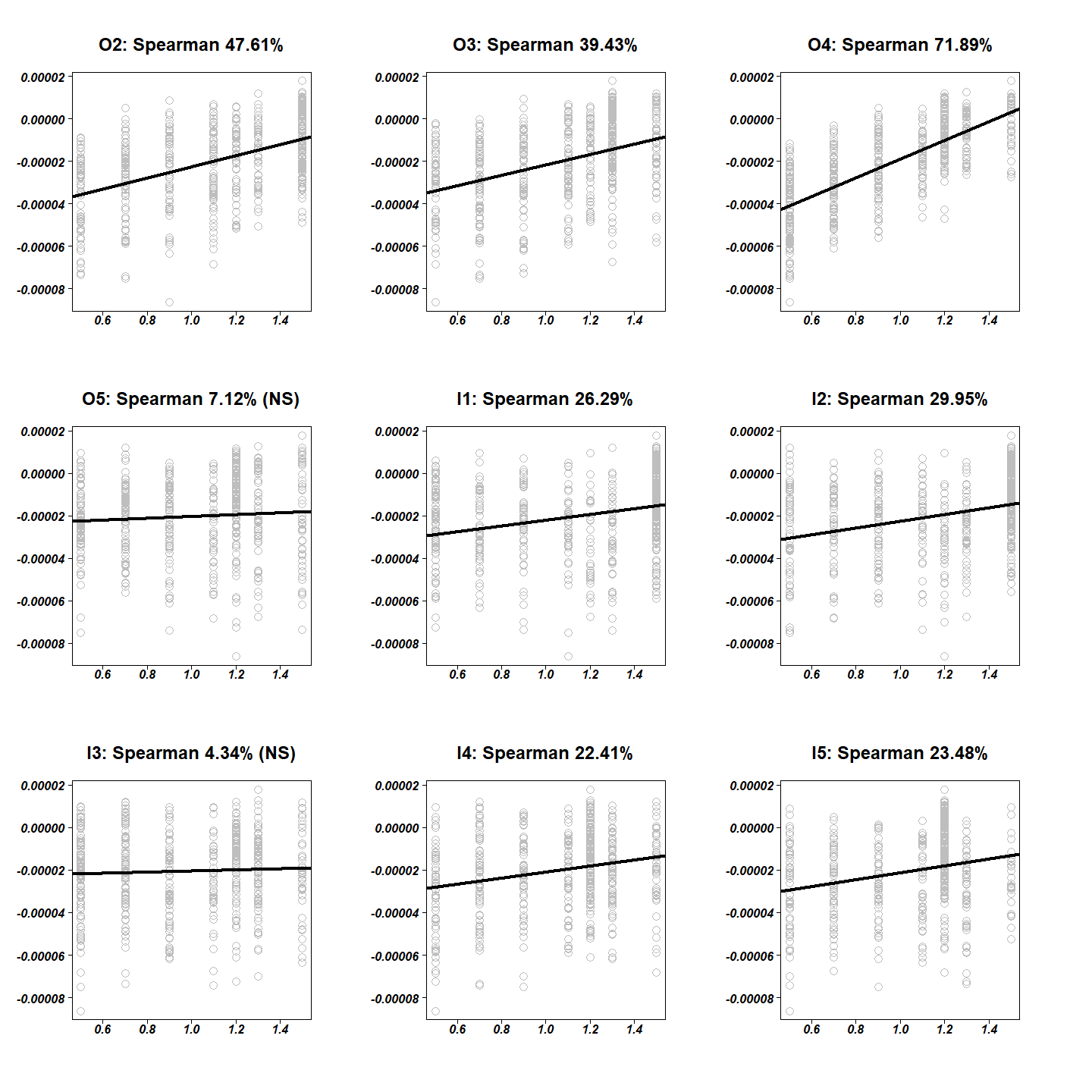

Supplement: Supplementary file 3 — Supplementary Information 3. [file 41598_2024_63077_MOESM3_ESM.tiff]

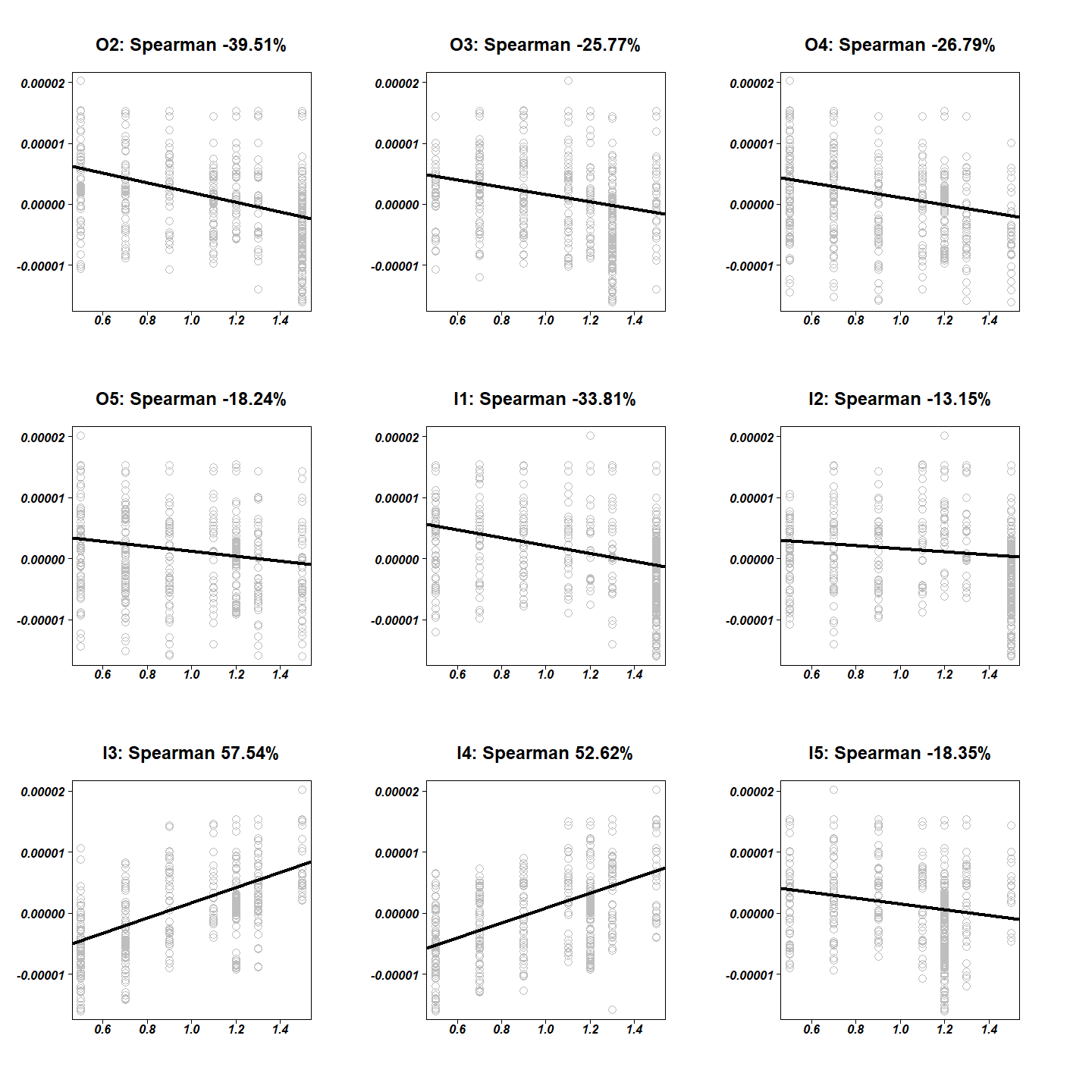

Supplement: Supplementary file 4 — Supplementary Information 4. [file 41598_2024_63077_MOESM4_ESM.tiff]

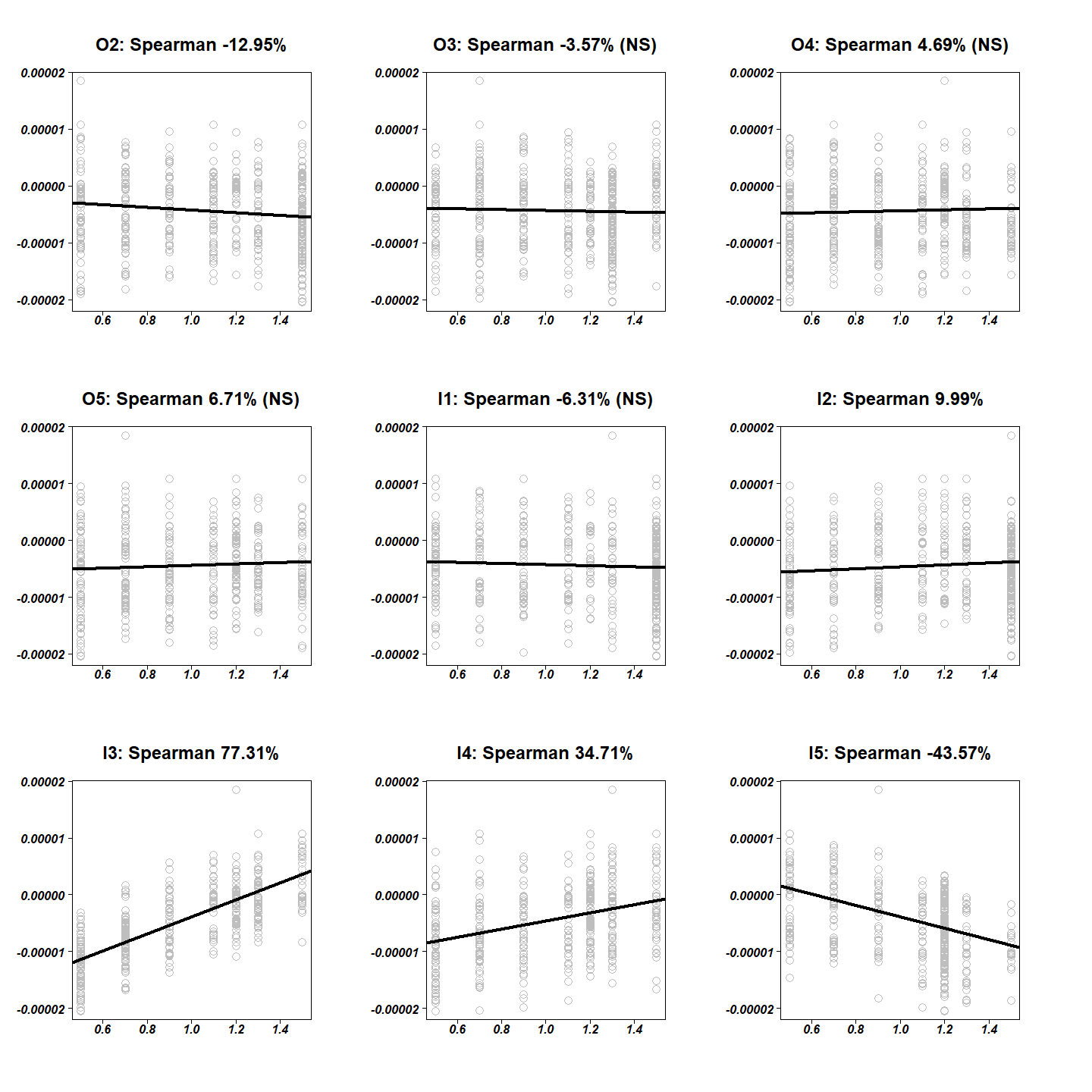

Supplement: Supplementary file 5 — Supplementary Information 5. [file 41598_2024_63077_MOESM5_ESM.tiff]
