# Supplementary material for: Increased stability of short femoral stem through customized distribution of coefficient of friction in porous coating
Source: Sci Rep. 2024 May 28;14:12243. doi: 10.1038/s41598-024-63077-w (PMC11133419; doi:10.1038/s41598-024-63077-w)
Supplement: Supplementary file 6 — Supplementary Information 6. [file 41598_2024_63077_MOESM6_ESM.docx]

**Supplementary information:**

**Supplementary File 1.** Validation of FEA model through comparison to the experimental model. (a) Exterior surface maximal principal strains in lateral cortex from the experiment measured with DIC; (b) Exterior surface maximal principal strains in lateral cortex from the FEA model; (c) Boxplot of the strains in the lateral cortex of the experiment measured with DIC VS the FEA model (Both the DIC and FEA demonstrated similar strains, which were fluctuated within the same range); (d) Exterior surface maximal principal strains in medial cortex from the experiment measured with DIC; (e) Exterior surface maximal principal strains in medial cortex from the FEA model; (f) Boxplot of the strains in the medial cortex of the experiment measured with DIC VS the FEA model (Both the DIC and FEA demonstrated similar strains, which were fluctuated within the same range).

**Supplementary File 2.** Spearman’s correlations between difference in strains of proximal lateral area L1 and coefficients of friction in different porous coat areas (O2, O3, O4, O5, I1, I2, I3, I4, I5).

**Supplementary File 3.** Spearman’s correlations between difference in strains of proximal lateral area L2 and coefficients of friction in different porous coat areas (O2, O3, O4, O5, I1, I2, I3, I4, I5).

**Supplementary File 4.** Spearman’s correlations between difference in strains of proximal medial area M1 and coefficients of friction in different porous coat areas (O2, O3, O4, O5, I1, I2, I3, I4, I5).

**Supplementary File 5.** Spearman’s correlations between difference in strains of proximal medial area M2 and coefficients of friction in different porous coat areas (O2, O3, O4, O5, I1, I2, I3, I4, I5).
